# Sclerite-bearing annelids from the lower Cambrian of South China

**DOI:** 10.1038/s41598-019-40841-x

**Published:** 2019-03-20

**Authors:** Jian Han, Simon Conway Morris, Jennifer F. Hoyal Cuthill, Degan Shu

**Affiliations:** 1 Shaanxi Key Laboratory of Early Life and Environment, State Key Laboratory of Continental Dynamics, Department of Geology, Northwest University, 229 Taibai Road, Xi’an, 710069 P.R. China; 2 Department of Earth Sciences, University of Cambridge, Downing Street, Cambridge, CB2 3EQ UK; 3 Earth-Life Science Institute (ELSI), Tokyo Institute of Technology, Tokyo, 152-8550 Japan

## Abstract

Cambrian annelids are strikingly diverse and reveal important details of annelid character acquisition. Their contribution, however, to a wider understanding of the evolution of the trochozoans (encompassing the annelids as well as such groups as the brachiopods and molluscs) remains limited. Thus the early annelids had been linked to a variety of cataphract Cambrian metazoans, notably *Wiwaxia* and the halkieriids, but recent work assigns such fossils to stem-group molluscs. Here we report two new annelids from the Lower Cambrian Chengjiang Lagerstätte, South China. *Ipoliknus avitus* n. gen., n. sp. is biramous with neurochaetae and notochaetae, but significantly also bears dorsal spinose sclerites and dorso-lateral dentate sclerites. *Adelochaeta sinensis* n. gen., n. sp. is unique amongst Cambrian polychaetes in possessing the rod-like supports of the parapodia known as aciculae. This supports phylogenetic placement of *Adelochaeta* as sister to some more derived aciculate Palaeozoic taxa, but in contrast *Ipoliknus* is recovered as the most basal of the stem-group annelids. Sclerites and chaetae of *I*. *avitus* are interpreted respectively as the remnants and derivatives of a once more extensive cataphract covering that was a characteristic of more primitive trochozoans. The two sets of chaetae (noto- and neurochaetae) and two sets of sclerites (spinose and dentate) suggest that in a pre-annelid an earlier and more complete scleritome may have consisted of four zones of sclerites. Other cataphract taxa from the Lower Palaeozoic show a variety of scleritome configurations but establishing direct links with such basal annelids as *Ipoliknus* at present must remain conjectural.

## Introduction

Along with the deuterostomes and ecdysozoans, the spiralians^1,2^ (that broadly encompass the lophotrochozoans^3^) are one of the three principal phylogenetic pillars of the Bilateria and are notable for a diversity of body plans. Many of the inter-relationships amongst the spiralians are still controversial, but following Kocot^2^ the lophotrochozoans can be broadly divided into the platyzoans (probably paraphyletic^4^) and the trochozoans (whose roster usually included the annelids, brachiopods, molluscs, nemerteans, and phoronids but less certainly one or other “polyzoan”). Amongst this latter assemblage, the phylum Annelida, and notably the paraphyletic assemblage of polychaetes, are important both in range of anatomical form and ecologies^5^. Yet many aspects of their evolution remain controversial. To some extent this applies to their internal relationships^6–10^, but much more so in terms of their origins and thus their possible connections to other trochozoan phyla. In this latter context most notable are the potential links to the brachiopods and molluscs. Thus, despite the growing roster of stem-group annelids^11–15^ that have been recorded from a number of Cambrian Fossil-Lagerstätten^16–23^, the connections of these disparate taxa to what have generally been identified as the most primitive of extant families^6–10^ are not obvious^11^. Moreover, thumb-nail sketches of the purported appearance of the ancestral annelid^6,7,24^ find few obvious counterparts amongst Cambrian polychaetes^12^, although here allowances need to be made for the potentially low fossilization potential of critical features^25^.

In attempting to establish the course of early trochozoan evolution, including possible links between the annelids and other phyla, various workers have looked to a variety of lower Palaeozoic sclerite-bearing (cataphract) metazoans^26–37^. In this regard key taxa include *Calvapilosa*^28^, *Halkieria*^26,31^ and related forms^29^, *Orthrozanclus*^32,33^, *Oikozetetes*^34,37^ and *Wiwaxia*^21,27,35,36^. The broad consensus remains (albeit with dissenting voices^26,38^) that these taxa are molluscan, and specifically should be assigned to stem-group aculiferans^28–30,35,39^. Nevertheless, the exact relationships between these taxa, as well as other scleritomic groups such as the tommotiids^40–45^, remain open to debate. For example, one reappraisal of *Halkieria* and *Orthrozanclus* confirms their close relationship but also contrary to the consensus argues for a relationship to the camenellan tommotiids^33^. More precise comparisons are somewhat frustrated because the latter group are effectively known only from disarticulated sclerites. Nevertheless, this proposed connection would in turn suggest that *Halkieria* and *Orthrozanclus* are phylogenetic neighbours of the trochozoan brachiopods and phoronids^40,43,44^ rather than the molluscs. The phylogenetic position of *Wiwaxia* has also been contested^27,36^, with conflicting interpretations including a relative proximity to either the annelids^38^ or molluscs, although the latter remains the current consensus^28^.

The various attempts to place these taxa in putative stem-groups also depend on which features could be plesiomorphic amongst two or more trochozoan phyla. Any such assignment in turn has implications for the likely mode of life of putative ancestors, not least style of feeding and relative motility^1^. It is likely, however, that whether sessile or motile (and if so possibly slug-like) the common ancestors of the annelids, brachiopods (plus phoronids), and molluscs possessed some sort of scleritome with a chitinous composition. If the descendant forms are any guide it is likely that this original scleritome consisted of several distinct zones. Subsequently as part of the trochozoan radiation into ultimately distinct phyla, this ur-scleritome underwent major changes in the distribution and morphology of the component sclerites, as well as in many cases subsequent and independent mineralization.

**Figure 1:**
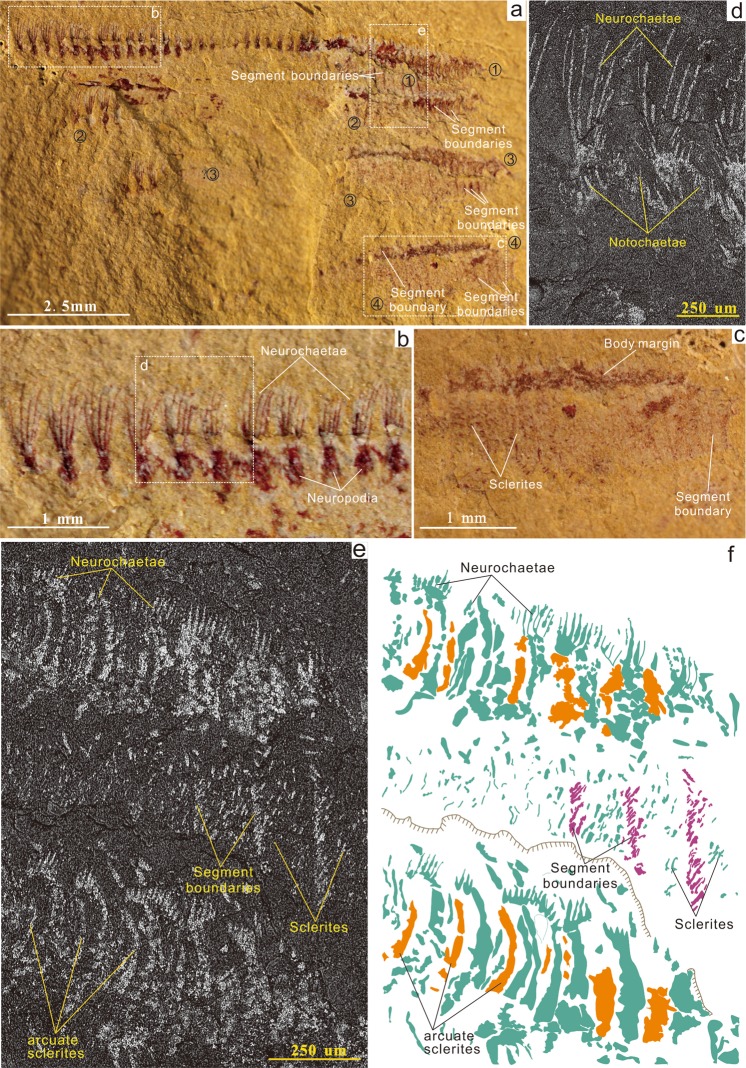
The annelid *Ipoliknus avitus* gen. et sp. nov. (ELI-EC051) from the Lower Cambrian Chengjiang Lagerstätte. (**a**) Four specimens (1–4), partially overlapping and sub-parallel with boxes showing locations of (**b**), (**c**) and (**e**). (**b**,**d**) Detail of right anterior showing biramous parapodia with neurochaetae and notochaetae in white light (**b**) and close-up in back-scatter electron (BSE) imagery (**d**). (**c**,**e**) Dorsal segments with sclerites converging on the midline and arcuate dentate-bearing sclerites alternating with apparently unarmoured sclerites in white light (**c**) and BSE (**e**). (**f)**, interpretative sketch of (**e**) showing the location of arcuate sclerites (yellow), dorsal sclerites, chaetae, etc.

**Figure 2:**
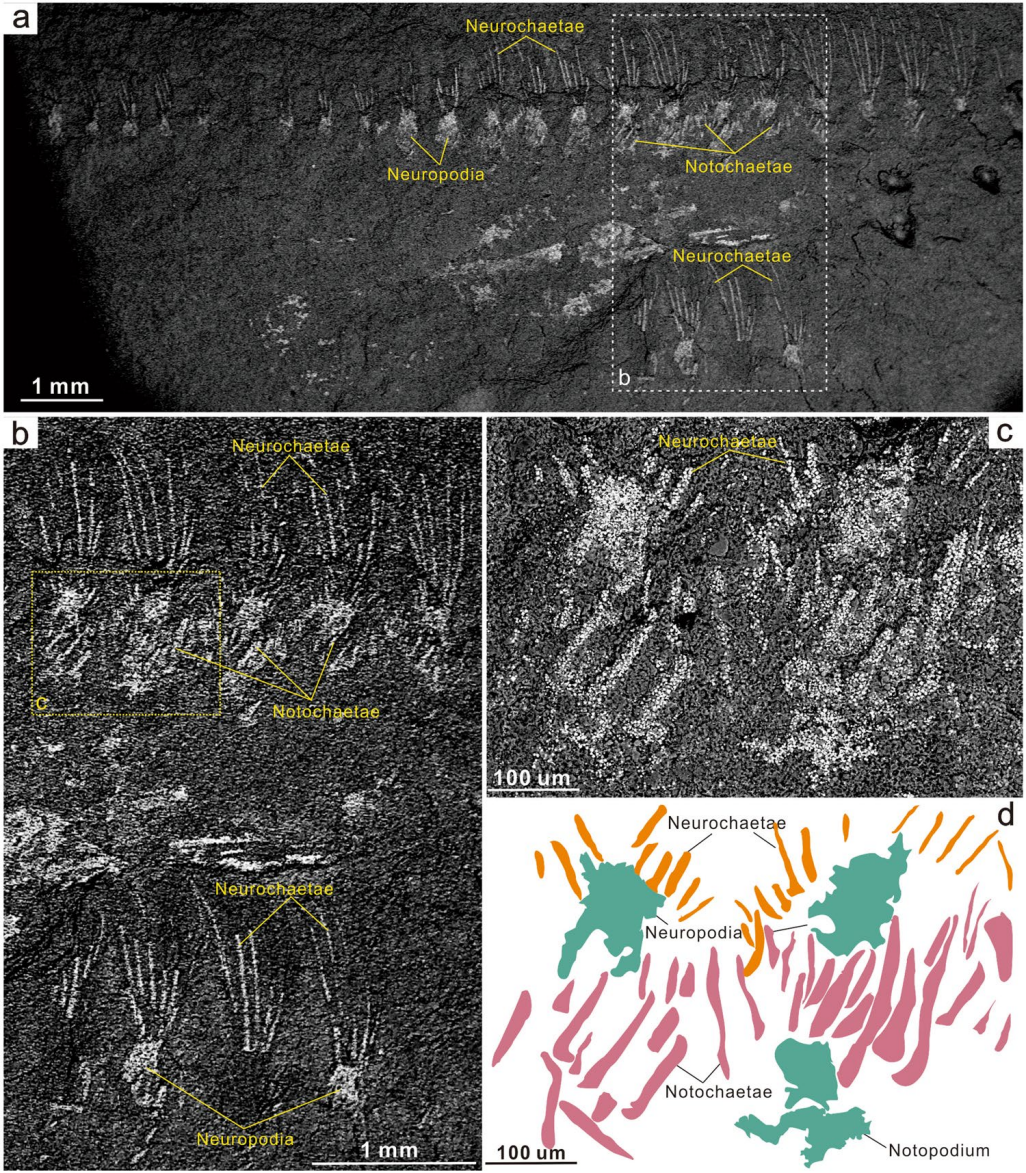
Back-scatter electron micrographs of sclerites in *Ipoliknus avitus* gen. et sp. nov. (ELI-EC051). (**a**) Overview with box indicating location of (**b**). Details of the chaetae and box indicating location of (**c**). (**c**) Close-up of massive notochaetae and illustration of preservation as framboidal pyrite. (**d)** Interpretative drawing of (**c**); neurochaetae, notochaetae, neuropodium and notopodium are colour-coded.

**Figure 3:**
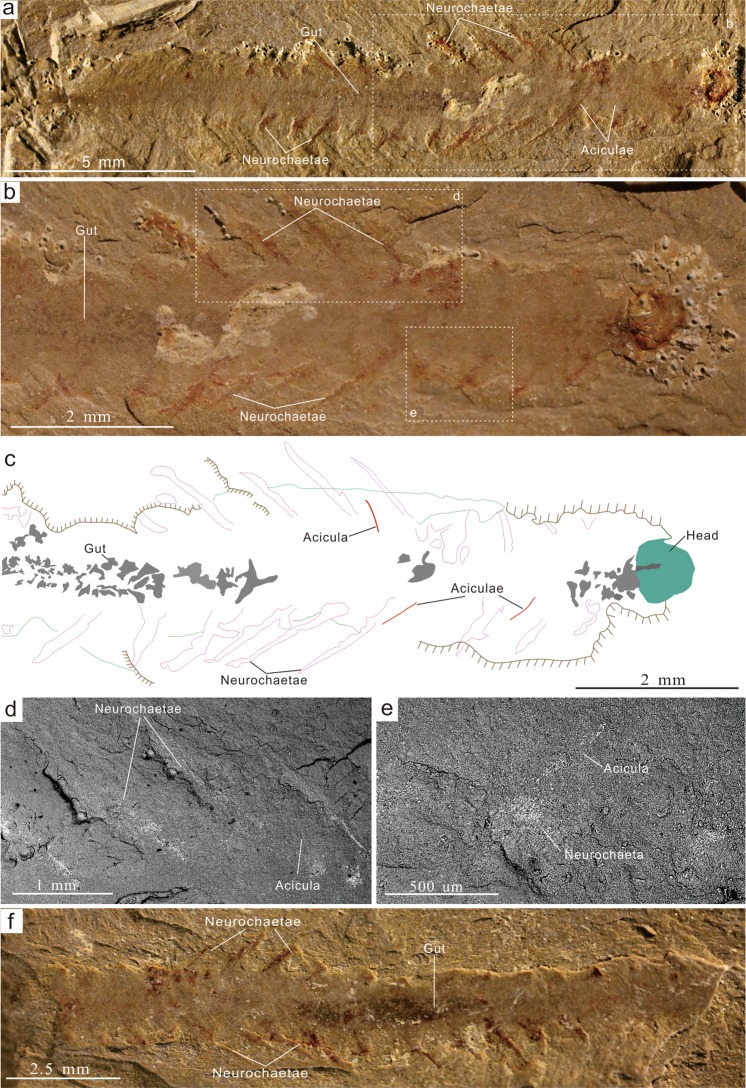
The annelid *Adelochaeta sinensis* gen. et sp. nov. (**a**–**e**) Holotype ELI -JS1136. (**a**) Part, nearly complete specimen showing segmented body with narrow bundles of neurochaetae and head. (**b**) Anterior region and boxes indicating location of (**d**) and (**e**). (**c**), interpretative drawing of (**b**) showing the disposition of possible aciculae. (**d**,**e**) back-scatter electron (BSE) imagery of left (**d**) and right (**e**) margins showing neurochaetae and associated aciculae. (**f**) Counterpart of holotype.

**Figure 4:**
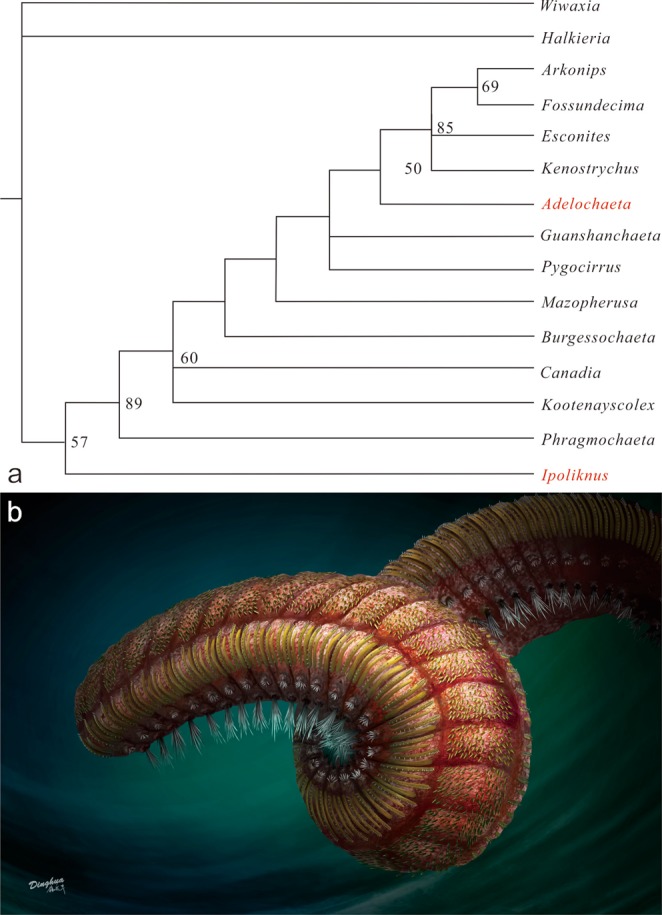
(**a**) Strict consensus phylogeny of fossil polychaetes. Parsimony analysis of new genera *Ipoliknus* and *Adelochaeta* (in red) plus 13 other fossil taxa^10,11^, for 24 morphological characters (18 parsimony informative; Character Appendix), recovered 4 most parsimonious trees of length 30, with consistency index CI = 0.70 and retention index RI = 0.81. Node labels show the bootstrap support value. (**b**) Reconstruction of *Ipoliknus avitus*.

**Figure 5:**
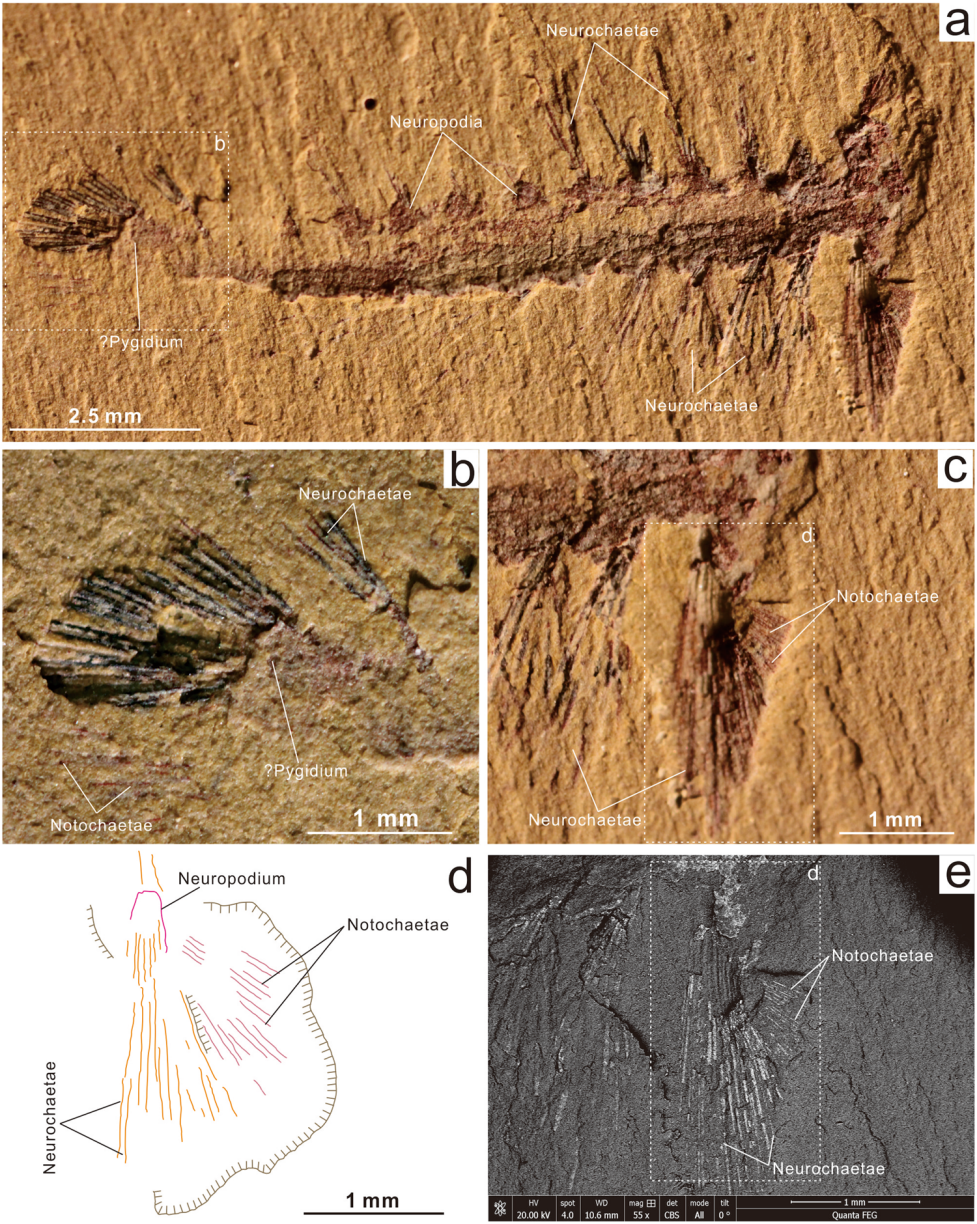
Undetermined annelid (ELI-JS044A,B) from the Lower Cambrian Chengjiang fauna. (**a**) ELI-JS044A, body with ?pygidium and box indicating location of (**b**). (**b**) Close-up of the ?pygidium with chaetae. (**c**) First right parapodium with fan-shaped neurochaetae and notochaetae. (**d**) Camera-lucida drawing of the first right parapodium. (**e**) Back-scatter electron (BSE) image of (**c**) and box indicating location of (**d**).

**Figure 6:**
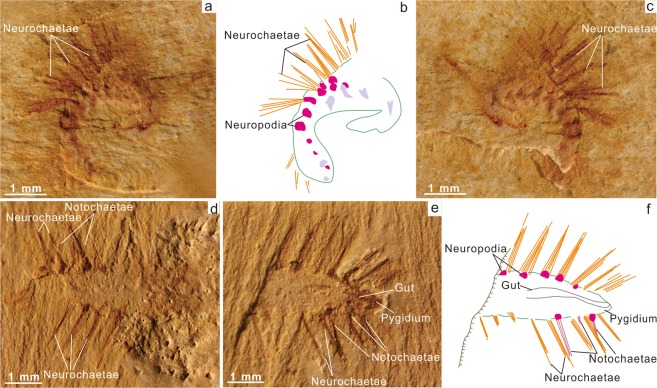
Undetermined annelids from the Lower Cambrian Chengjiang fauna. (**a–c**) ELI-EJ050A,B, coiled specimen (part and counterpart) with prominent bundles of chaetae. (**b)** Interpretative drawing of (**a**). (**d**–**f**) ELICJ181A, B. incomplete specimen (part and counterpart), posterior section with narrow bundles of neurochaetae and notochaetae. Gut visible towards posterior termination. (**f)** Interpretative drawing of (**e**).

As a contribution to unravelling the early stages of trochozoan evolution, we describe a new polychaete, *Ipoliknus avitus* n. gen, n. sp. (Figs 1, 2, 4b), from the Lower Cambrian Chengjiang Lagerstätte of South China. In addition to its canonical notochaetae and neurochaetae, this worm also possesses dorsal and dorso-lateral sclerites. The discovery of this basal polychaete (Fig. 4a) indicates an evolutionary link of the annelids to at least some of the other sclerite-bearing trochozoans. In addition, from co-eval deposits we describe another polychaete, *Adelochaeta sinensis* n. gen., n. sp. (Fig. 3). This new taxon is apparently unique amongst Cambrian forms in its possession of internal cuticular rods known as aciculae. Amongst Palaeozoic polychaetes *Adelochaeta* is more derived and the presence of aciculae supports a position close to some other aciculate taxa (Fig. 4a) (although a somewhat more basal position is recovered using Bayesian analysis under a likelihood model of character evolution, Supplementary Information Fig. 3). Finally, three other specimens appear to be distinct from these two taxa but with the limited information available they remain in open nomenclature (Figs 5, 6).

## Results

### Systematic palaeontology. Superphylum Lophotrochozoa

Phylum Annelida Lamarck 1802^46^.

Class Polychaeta Grube 1850^47^.

Family Ipoliknidae n. fam.

*Ipoliknus* Han, Conway Morris and Shu 2019 n. gen.

*Ipoliknus avitus* Han, Conway Morris and Shu 2019 n. sp.

#### Remarks

A single specimen of a polychaete^19^ has been described from the nearby and slightly younger Guanshan Fossil-Lagerstätte, but with *Ipoliknus*, *Adelochaeta*, at least two forms in open nomenclature and possible sipunculans^48^ it is evident that despite their scarcity annelidan diversity in the Chengjiang Lagerstätte was notable and perhaps second only to the Burgess Shale^16,21^.

#### Etymology

Generic name from Greek for worm (*ipos*) and fan (*liknos*), latter with reference to prominent neurochaetae. Specific name from Latin for ancestral (*avitus*).

#### Diagnosis

Body centimetric, relatively broad (c. 1.5 mm), c. 50 segments, biramous parapodia. Neurochaetae prominent, c. 6 chaetae per bundle. Notochaetae massive. Dorsum bears two types of sclerites. Abaxially prominent elongate sclerites with dentate margin (c. 500 µm) while straddling midline an array of spinose sclerites (c. 150 µm). Head not known.

#### Type material

ELI-EC051A, B [1] (Figs 1a,b,d,e; 2), holotype; ELI-EC51 A, B [2–3] (Figs 1a,c,e; 2a,b), paratypes, all specimens comprise parts and counterparts, preserved in shale as a “pop-out”, weathered with abundant associated framboidal and more massive pyrite.

#### Provenance

Qiongzhusi (Chiungchussu) Formation, Yu’anshan Member (*Eoredlichia* zone), Cambrian (Series 2 (Stage 3)). All material is from the Ercai section, Haikou, Kunming, Yunnan province.

Family Adelochaetidae n. fam.

*Adelochaeta* Han, Conway Morris and Shu 2019

n. gen.

*Adelochaeta sinensis* Han, Conway Morris

and Shu 2019 n. sp.

#### Etymology

Generic name from Greek for faint or dim (*adelos*), referring to indistinctness of the chaetae. Specific name a reference to its Chinese location.

#### Diagnosis

Robust body (c. 18 mm), c. 20 segments. Narrow bundles of biramous chaetae, shorter posteriorly.

#### Holotype

ELI**-**J1050 A, B (Fig. 3).

#### Provenance

Qiongzhusi (Chiungchussu) Formation, Yu’anshan Member (*Eoredlichia* zone), Cambrian (Series 2 (Stage 3)). Material is from the Jianshan section, Haikou, Kunming, Yunnan province.

#### Descriptions

Material is rare, consisting of a single specimen of *Adelochaeta* and four of *Ipoliknus*. The latter is an association that shows sub-parallel arrangement and partial overlap (Figs 1a; 2a,b)), an orientation most likely the result of current activity. All specimens show a preservation typical of the Chengjiang Lagerstätte^25,48^. Some regions of the body in *Ipoliknus* (notably chaetae and sclerites) are pyritized. Now oxidized, the relict framboids occur as both scattered patches on the trunk and more particularly concentrated along the sclerites, chaetae and associated parapodia (Fig. 2c). Elemental analyses of *Ipoliknus* confirm the abundance of iron, but other major elements are not enhanced (Supplementary Information Fig. 1).

*Ipoliknus* (Fig. 4b) has a relatively broad trunk, composed of c. 50 segments, with biramous parapodia (Figs 1b,d,e; 2a,b). Neurochaetae are prominent, each forming a fan of c. 6 chaetae that project more or less at right angles from the body. Each chaetal fascicle converges on a capsule-like structure (Figs 1b,d; 2b). Notochaetae are massive, arranged sub-parallel to the longitudinal axis (Figs 1d,e; 2b,c). Neither head nor pygidium is visible. Segment boundaries are evident (Fig. 1c,e), but there is no direct evidence for internal anatomy.

*Ipoliknus* is noteworthy for its two sets of cuticular structures (Fig. 1c,e–f). Abaxial to the midline are gently arcuate sclerites (c. 500 µm), separated by narrow zones of cuticle. Some possess a prominent dentation, but intervening sclerites may have lacked denticles. The other type of sclerite occupies the dorsum and is represented by large numbers of small sclerites (c. 10 µm long). Each sclerite has a relatively broad base and an even degree of taper. They lie oblique to the longitudinal axis of the trunk, converging on the midline. Given that both these sclerite types show the same sort of preservation as the chaetae, most likely they also were composed of chitin.

*Adelochaeta* differs in many respects from *Ipoliknus*. The former (Fig. 3) is characterized by a broad and smooth trunk, consisting of c. 20 segments. A defined head is relatively large but yields no information on possible palps/antennae or jaws. Arising from the body the notochaetae appear to have been short and relatively few in each fascicle. The neurochaetae are thin, forming posteriorly directed narrow bundles. Adaxial to the chaetae are internal elongate rod-like structures (Fig. 3c–e). In common with the chaetae they are not particularly well preserved, but are clearly distinguished from the adjacent body. Their location is consistent with them being equivalent to the aciculae that characterize some, but not all, crown group polychaetes. An alimentary canal is also preserved.

### Other polychaetes

In addition to these two new polychaetes three additional specimens are available (Figs 5, 6, Supplementary Information Fig. 2). Whilst similar to *Ipoliknus*, the chaetae in these specimens are conspicuously longer and they may represent at least two new taxa. ELI-JS044 (Fig. 5; Supplementary Information Fig. 2) has a straight orientation and shows 14 segments. ELI-EJ050 (Fig. 6a–c) is quite tightly coiled; such a disposition could have resulted from pre-mortem stress. ELI-CJ181 (Fig. 6d–f) is incomplete, displays 11 segments, lacks the anterior but shows evidence for the pygidium, albeit without evidence for cirri or other extensions. In all these specimens the elongate chaetae are probably the neurochaetae, while in ELI-JS044 also visible is a fan of finer notochaetae subparallel to the body (Fig. 5b–e). In contrast specimen ELI-CJ181 (Fig. 6d–f) shows more elongate notochaetae, sub-parallel to the neurochaetae. The only detail of the internal anatomy is a gut trace in ELI-CJ181. Pyritization is extensive in ELI-JS044, and although there is a relative enrichment in copper, its coincidence with the iron (Supplementary Information Fig. 2) suggests an association with the iron pyrites (as a chalcopyrite).

### Evolutionary relationships

Recent phylogenetic analyses of the annelids^6–10^ have been augmented by those that include a variety of fossil taxa^12,14,15^. These new phylogenies^14,15^ represent important advances, but indicate comparatively little morphological character overlap between extant species and Cambrian fossil taxa, which generally preserve far fewer soft-tissue or micro-scale characters. Because of the recognition of sclerites in *Ipoliknus* our principal aim here is to place these new fossils in the context of early annelid evolution. Accordingly our main analysis (Fig. 4a) encompasses *Adelochaeta* and *Ipoliknus* as well as those fossil taxa previously considered^14,15^.

This analysis results in a well-resolved phylogeny that is broadly congruent with the most recently published phylogeny^15^. A parallel Bayesian phylogenetic analysis recovers a broadly similar topology (Supplementary Information Fig. 3), although *Adelochaeta* is recovered in a somewhat more basal position within the annelids. With the tentative exception of *Guanshanchaeta* (ref.^19^, Fig. 2c), *Adelochaeta* is evidently unique amongst Cambrian polychaetes in possessing aciculae (Fig. 3c–e). In the most parsimonious phylogenies (Fig. 4a) this genus is sister to some more derived taxa from the later Palaeozoic. Aciculae are taken as a key character in the eponymous Aciculata^5,12,14^, and this could support such a placement for *Adelochaeta*. Nevertheless, given the paucity of available character states caution is necessary and the presence of aciculae in *Adelochaeta* may be convergent. Support for this possibility comes from the scattered occurrences of aciculae in such groups as the psammodrilids and apistobranchids^5^ that phylogenetically lie well aside the Aciculata. In terms of our proposed phylogeny the Sirius Passet Lagerstätte *Phragmochaeta*^17^ remains near basal^14^. Most significantly, however, *Ipoliknus* is revealed to be basal-most of the annelids and here the sclerites seem to be of particular note. Apart from possibly similar structures that sometimes co-occur with the disarticulated chaetae in the lower Cambrian *Baltichaeta*^22^, these sclerites find no counterpart in the integuments of any other fossil or extant polychaete. In particular, although a number of annelids bear dense fields of recurved chaetae^49^ these are of neuropodial origin and unrelated to the sclerites of *Ipoliknus*. So too, it is important to emphasize that these sclerites are quite distinct from the calcitic plates of the geologically younger machaeridian annelids^12,50^, be it in terms of composition, distribution and mode of growth. Given, therefore, that within the annelids these sclerites are unique to *Ipoliknus*, there would appear to be broadly two alternatives. The first is that they are an example of evolutionary convergence. An alternative view, consistent with the basal-most phylogenetic position of *Ipoliknus* (Fig. 4a), is that these structures are the remnants of a once more complete cuticular coating that otherwise characterized more primitive cataphract trochozoans.

The recognition of an annelid with a scleritome invites brief consideration of the possible significance of *Ipoliknus* in the wider schema of metazoan evolution, specifically the Trochozoa which form an apparently monophyletic assemblage within the larger Lophotrochozoa^3,51^. So far as the trochozoans are concerned this group includes annelids, as well as brachiopods, molluscs, nemerteans and phoronids. Other phyla are sometimes included but are not of immediate relevance to our observations. Amongst the principal trochozoan phyla there are a variety of rival groupings, but there is generally strong support for brachiopods and phoronids being a sister-group (and with a possible further link to the nemerteans^52^), and some evidence that they in turn may be related to an annelid-mollusc clade. The marked disparity of these phyla and a scarcity of synapomophies (or alternatively character states that exhibit contradictory distributions amongst the various groups) means that the fossil record is best placed to provide potential information on transitional forms. In this context a diversity of taxa possessing various types of scleritome (albeit with the curious exception of *Odontogriphus*^39^) have been a focus of attention as candidate stem-groups for one or other of the trochozoan phyla. Notable in this respect are the Cambrian taxa *Wiwaxia*^21,27,35,36,39^, *Halkieria*^26,31^, *Orthrozanclus*^32,33^, *Oikozetetes*^34,37^, *Sinosachites*^29^, and from the Ordovician *Calvapilosa*^28^. The relationships of these taxa, both with respect to each other and also more widely, are not fully resolved^33,38,53^. Currently most, if not all, are regarded as early molluscs^30,53^, specifically stem-group aculiferans that broadly pre-figure a polyplacophoran-like arrangement^28,35^. More recently, however, a description^33^ of a new orthrozanclid (*O*. *elongata* from the Chengjiang Lagerstätte) has questioned this consensus, albeit without the benefit of a cladistic analysis. In this reinterpretation *Orthrozanclus* and *Halkieria* are confirmed to be closely related^32^, but this analysis rejects a close connection between these two taxa and the wiwaxiids. It does, however, argue for a close affinity between these halkieriids and a group of tommotiids known as the camenellans^45^. This latter group has in turn been convincingly linked, by forms such as *Micrina* and *Eccenthrotheca*, to the brachiopods and phoronids^40–44^. In this new formulation, *Wiwaxia* would be moved more basal-wards and so could be re-interpreted as a stem-group trochozoan^27,38,54^.

In discussions of the nature of the early trochozoans a key feature concerns the chitinous chaetae. Best known in the annelids and brachiopods^55^, they find occasional counterparts elsewhere amongst the trochozoans. Amongst fossil material, one notable example concerns the coiled shell *Pelagiella*^56^ which is evidently a mollusc but of uncertain position. Amongst extant molluscs chaetae are known in some juvenile octopods^57^. Employment of narrow cellular extensions, the microvilli, for the secretion of not only these various sorts of chaetae but also other types of sclerites is interpreted as a plesiomorphy of the Trochozoa^21,27^, although these fundamental homologies may be less obvious given the variety of sclerite form, such as their being hollow or mineralized^35^. So too the details of scleritome configuration show wide variation, even in apparently quite closely related taxa. Thus although *Halkieria* and *Orthrozanclus* are both assigned to the Halkieriidae^32,33^, comparisons between their scleritomes reveal important and intriguing differences. In these two taxa not only are there respectively two and one terminal shells, but the shared tripartite arrangement of the sclerite zones (basal, mid, upper) is such that the basal-zone sclerites in *Orthrozanclus* are strikingly similar in terms of morphology and orientation to those of the mid-zone in *Halkieria*^33^. This otherwise unremarked mosaicism and the possibility that various microstructures within the sclerites could be convergent^53^ suggest that the details of early trochozoan evolution may require further discoveries.

In this context it is important to note that the sclerites of *Ipoliknus* find no exact counterpart amongst other known trochozoans in terms of their specific shape. Nevertheless the description of this new annelid allows for two potentially important observations. First, given the preservation of the sclerites is identical to the adjacent chaetae this suggests that they too were not only chitinous but presumably also microvillous in their construction. By implication both chaetae and sclerites would be homologous with the various types of sclerites that characterize a number of other trochozoans. Second is the observation that the sclerites on the dorsal surface of *Ipoliknus* are arranged in two zones (Fig. 1e,f), while adaxially there are two zones of chaetae (neurochaetae and notochaetae, Fig. 1d). This hints that forms ancestral to *Ipoliknus* may not only have had a scleritome^58^, but one composed of four distinct zones.

If there was such a sclerite configuration, then in the lineage that led to the annelids the two ventro-lateral zones would have ultimately given rise to the biramous arrangement of neurochaetae and notochaetae. These were deployed respectively as locomotory units and a more dorsal thatch-like arrangement that conferred protection^14,24,26^. In an already widely accepted scenario^6,11,24^ the transition to a fully fledged annelidan body plan^58^ was driven by (a) enhanced motility and, in combination with a body undulation, the development of a stepping pattern of metachronal levers (the parapodia and neurochaetae acting as points d’appui) and (b) in post-*Ipoliknus* forms (Fig. 3a) the loss of the two zones of dorsal sclerites with any protective role being now adopted by the notochaetae^13,17^.

## Conclusion

The Ediacaran taxon *Kimberella*^59^ has been regarded as a mollusc^30^, but has been alternatively placed as a more basal trochozoan^60^. In any event, along with the other bilaterians this group was probably beginning to diversify in the latest Proterozoic^60^. It is possible, however, that the principal radiations of trochozoans were an integral part of the Cambrian “explosion”. This expansion led not only to a remarkable diversity of animal forms, including the exceptional disparity seen amongst the Cambrian annelids. In this respect the range of taxa encompasses both the sclerite-bearing *Ipoliknus* and apparently aciculate *Adelochaeta*, as well as such taxa as different as *Phragmochaeta*, *Insolicorypha* and *Canadia*^12,16,17,21^. In passing, it may be worth noting that this latter taxon not only has especially well developed notochaetae and neurochaetae but also an inter-ramal gill that is intriguingly reminiscent of the molluscan ctenidia^16^. Such evolutionary mosaicism is an integral (if unduly neglected) feature of all adaptive radiations and also a reminder that the anatomical and genomic differences amongst the stem-group trochozoans that shortly led to groups as disparate as the annelids and molluscs would have been minimal. So too the sclerites of *Ipoliknus* point to links to other sclerite-bearing taxa, but the unravelling of this complex history must await new discoveries amongst the Cambrian Fossil-Lagerstätten.

## Methods

Specimens were observed using a binocular microscope Leica M80 complemented with interpretative camera-lucida drawings. Photographs were taken using a Canon 5D Mark III. Energy-dispersive spectroscopy (EDS) analysis and back-scatter electron (BSE) imagery without coating were conducted on an environmental scanning electron microscope (SEM) of FEI Quanta 400 FEG under low vacuum and 20 kv with an EDS system at the State Key Laboratory of Continental Dynamics, Northwest University, China.

## Supplementary information

Phylogeny and Energy dispersive spectroscopy of Chengjiang annelids

## Data Availability

Specimens are accessioned in the Shaanxi Key Laboratory of Early Life and Environment, Northwest University, Xi’an.
